# The duty of confidentiality during family involvement: ethical challenges and possible solutions in the treatment of persons with psychotic disorders

**DOI:** 10.1186/s12888-022-04461-6

**Published:** 2022-12-20

**Authors:** Kristiane Myckland Hansson, Maria Romøren, Bente Weimand, Kristin Sverdvik Heiervang, Lars Hestmark, Elleke G. M. Landeweer, Reidar Pedersen

**Affiliations:** 1 Centre for Medical Ethics, University of Oslo, Postboks 1130 Blindern, 0318 Oslo, Norway; 2 Center for Mental Health and Substance Abuse, Faculty of Health and Social Sciences, University of South-Eastern Norway, Drammen, Norway; 3 Division of Mental Health Services, Akershus University Hospital, Sykehusveien 25, 1474 Nordbyhagen, Norway; 4 University Center of Medical Sciences, University of Groningen, Antonius Deusinglaan 1, 9713 AV Groningen, The Netherlands

**Keywords:** Confidentiality, Ethics, Ethical challenges, Family involvement, Severe mental illness, Facilitators, Health services research

## Abstract

**Background:**

Family involvement during severe mental illness is still poorly implemented, contrary to evidence-based recommendations. Confidentiality issues are among the most prominent barriers, with mental health professionals facing complex ethical, legal, and practical challenges. However, research focusing on this barrier is very sparse. Nested within a cluster-randomised trial to implement guidelines on family involvement for persons with psychotic disorders in community mental health centres, the aim of this sub-study was to explore ethical challenges related to the duty of confidentiality as experienced by mental health professionals, and to explore key measures that might contribute to improving the handling of such challenges.

**Methods:**

In total 75 participants participated in 21 semi-structured focus groups, including implementation team members at the initial and late phase of the intervention period and clinicians who were not on the implementation teams, at late phase of implementation. We used purposive sampling and manifest content analysis to explore participants’ experiences and change processes.

**Results:**

Ethical challenges related to the duty of confidentiality included 1) Uncertainty in how to apply the legislation, 2) Patient autonomy versus a less strict interpretation of the duty of confidentiality, 3) Patient alliance and beneficence versus a less strict interpretation of the duty of confidentiality, 4) How to deal with uncertainty regarding what relatives know about the patients’ illness, and 5) Relatives’ interests versus the duty of confidentiality. Measures to facilitate better handling of the duty of confidentiality included 1) Training and practice in family involvement, and 2) Standardisation of family involvement practices.

**Conclusion:**

When health professionals gained competence in and positive experiences with family involvement, this led to vital changes in how they interpreted and practiced the duty of confidentiality in their ethical reasoning and in clinical practice*.* Especially, the need to provide sufficient information to the patients about family involvement became evident during the study. To improve the handling of confidentiality issues, professionals should receive training in family involvement and confidentiality statutes followed by practice. Furthermore, family involvement should be standardised, and confidentiality guidelines should be implemented in the mental health services.

**Trial registration:**

ClinicalTrials.gov Identifier NCT03869177. Registered 11.03.19.

**Supplementary Information:**

The online version contains supplementary material available at 10.1186/s12888-022-04461-6.

## Background

Patients with psychotic disorders and their relatives are often not offered the family involvement and support they are entitled to [1, 2], despite decades of substantial research evidence on patient [3–5] and relative [2, 6] outcomes, recommendations in government policies worldwide [7–10], and numerous attempts to increase the uptake of family interventions in routine care [4, 11, 12]. Meaningful engagement of family members in treatment and decision-making processes is hampered by clinical, organisational, cultural, and historical barriers [1, 12–14]. Among such impediments, the research literature identifies confidentiality issues as a prominent barrier, portrayed as a complex and controversial area of clinical practice [15–18].

The complexity of information sharing is partly due to a lack of trust between stakeholders – patients, relatives, and health care personnel [14], who may have different expectations, needs, and concerns [19, 20]. Patients express a number of concerns about involving their family in treatment, such as uncertainty regarding disclosure of sensitive information, fear of losing control, or notions that involvement will burden their family or will not be useful [21]. Relatives often contribute to the care process in various (implicit) ways [10], they want their contributions to be recognised [22, 23], and they express a need for information and support from professionals [24, 25]. Fulfilling these roles can become more challenging if relatives are kept “out of the loop” [18], affecting their commitment to caring and the relationship with the professionals/services [26, 27]. However, relatives experience devaluation, neglect, and lack of involvement [15, 22, 28], suffer from high unmet needs for information [16, 29, 30], and experience repeated refusals from the services who frequently invoke the duty of confidentiality as justification for this [15, 18, 26, 27, 31, 32]. Furthermore, studies show that mental health professionals frequently experience that patients refuse to involve their relatives [16], often are reluctant to share information with families [17, 18], struggle to balance patients’ and relatives’ interests regarding disclosure [33], and fear that breach of confidence could potentially result in legal or disciplinary action [15, 16]. Health care professionals also lack appropriate training in family involvement and confidentiality statutes, while confidentiality policies and guidelines are often ambiguous and under-implemented [34].

These different expectations, needs and concerns may create ethical challenges for care professionals. In this study an “ethical challenge” is defined as a situation where there is doubt or disagreement about what is right or good [35]. In this paper we draw on Beauchamp and Childress’s four principles of biomedical ethics [36] because weighting the principles of respect for autonomy, beneficence, non-maleficence, and justice might be helpful when dealing with ethical challenges. The principle of respect for autonomy has in particular left its mark on current western confidentiality policies and practices [17]. Contemporary bioethics made a pivotal contribution in terms of strengthening the emphasis on patients’ autonomy by formulating the concepts of “capacity to consent” and “informed consent” [37]. For a consent to be informed, the patient must be adequately informed by health personnel to hold substantial understanding and not be controlled by others, while intentionally authorising a professional to do something that is specifically mentioned in the consent agreement [36]. In the present context, respecting autonomy means that patients with the capacity to consent are to decide themselves which confidential information can be shared and with whom. The duty of confidentiality is also strongly emphasised in professional ethics codes, and is considered vital for the alliance with the patient and thus beneficial for the patient.

In most countries, the duty of confidentiality is included in health care legislation, stating that health care professionals shall prevent others from gaining access to patient information that they become aware of as professionals [38]. With few exceptions, health information may be disclosed to others only to the extent that the patient consents regardless of how sensitive the information is. In practice this means that relatives as a main rule are not entitled to get information about the patient if the patient has not consented. However, regardless of patient consent, professionals are often given the possibility to share general information, and to listen and provide support to relatives is usually not considered a breach of confidentiality [10]. In Norway, the health services also have an obligation to provide training and supervision for relatives, especially if the relatives have extensive tasks as informal careers [39].

This paper reports findings from a sub-study nested within the IFIP-trial: Implementation of guidelines on Family Involvement for persons with Psychotic disorders in community mental health centres (CMHCs) [40, 41]. When investigating which factors affected the implementation and how, the duty of confidentiality was identified as a key barrier [42]. Consequently, we performed a separate in-depth exploration of confidentiality issues with a particular focus on the changes that transpired within the participants and at the units while the implementation progressed.

Research focusing explicitly on ethical challenges related to confidentiality and family involvement is lacking, as are explorations of how barriers to information sharing are resolved ethically in practice [18]. In particular, situations where competent patients refuse to involve their family constitute an unresolved grey area. The aim of this paper is to contribute to a better handling of confidentiality by addressing these research gaps. Its scope is limited to challenges related to disclosure of information to relatives. The following research questions guided the data collection and analysis: 1) “What ethical challenges do mental health professionals experience related to the duty of confidentiality in family involvement during the treatment of persons with psychotic disorders?” and 2) “What measures are experienced as helpful to improve the handling of such challenges?”.

## Methods

This article conforms to the “Standards for Reporting Qualitative Research (SRQR): 21-items checklist” [43] (Additional file 1).

### Study design and context

The cluster randomised IFIP trial employed a responsive evaluation design [44] including process and formative evaluations [45]. The knowledge was generated through exploration of various stakeholders’ views and by continuously engaging in dialogue with the participants and key stakeholders before and during implementation.

### Focus groups - participants and data collection

**Fig. 1:**
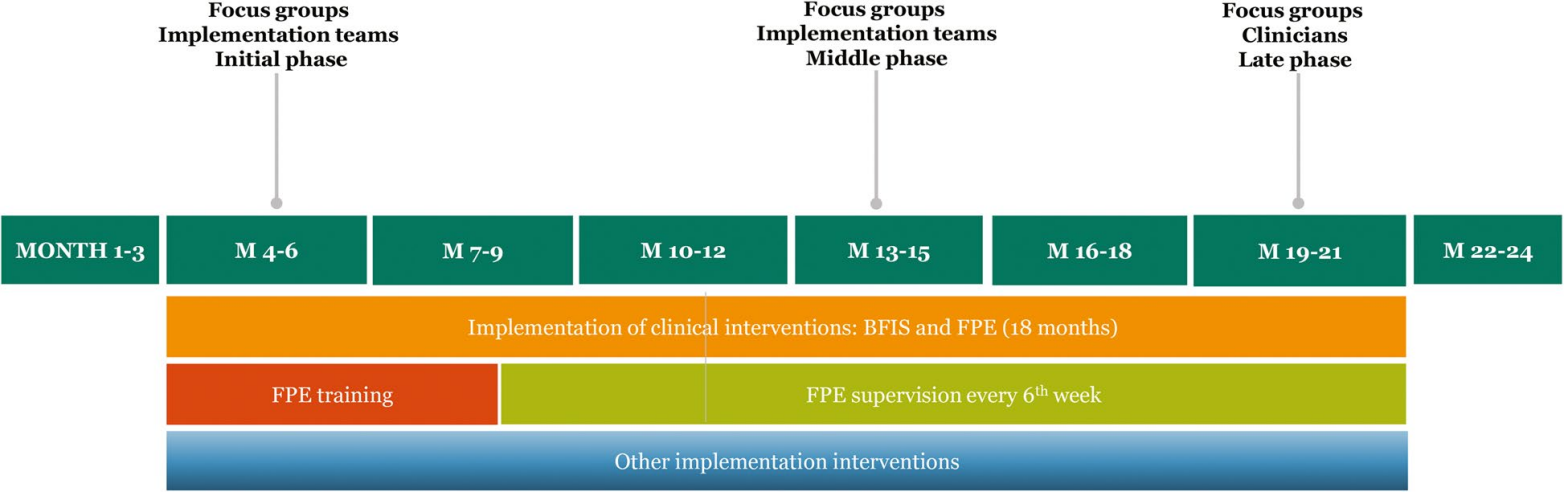
IFIP timeline BFIS: Basic family involvement and support FPE: Family psychoeducation

Each of the eight clinical sites in the experimental arm established a local implementation team of 3–8 persons who were responsible for the implementation at the unit. During the 18-month implementation period, we conducted 21 semi-structured focus groups with the implementation teams and clinicians (2019–2020). Implementation teams were interviewed twice at different stages of the implementation process. Figure 1 illustrates the data collection along with the IFIP implementation measures.

A purposive sampling strategy [46] followed naturally from the study design because we aimed at exploring experiences with confidentiality issues from participants engaged in the implementation work. When participating in the first round of focus groups (Fig. 1, initial phase), most implementation team members lacked competence and experience with family involvement. At the time of the second focus group (Fig. 1, middle phase), most had attended a four-day course in family psychoeducation (FPE), had received specific training in legal, ethical, and practical aspects of confidentiality, and were practicing family involvement. To expand on these accounts and learn from participants with less commitment to the implementation work, clinicians who were not part of the implementation teams were interviewed at a late phase of implementation (Fig. 1, clinicians). A total of 75 participants – including 67 clinicians and 8 unit managers – were included in the study, and 27 implementation team members participated twice (Table 1).

**Table 1:** Participant characteristics

Study sample	Implementation teams Months 2–3 of implementation (*N* = 38, 8 focus groups)		Implementation teams Months 9–10 of implementation (*N* = 39, 8 focus groups)		Clinicians Months 15–16 of implementation (*N* = 25, 5 focus groups)	
	***N***	%	***N***	%	***N***	%
**Sex**						
Male	6	16	5	13	5	20
Female	32	84	34	87	20	80
**Age**						
20–35	6	16	5	13	7	28
36–50	11	29	16	41	11	44
51–70	21	55	18	46	7	28
**Profession/ role**						
Section/unit manager	6	16	5	13		
Physician	4	11	3	8	4	16
Psychologist	5	13	5	13	16	64
Psychiatric nurse	14	37	15	38	1	4
Other	9	24	11	28	4	16

We developed three semi-structured interview guides adapted to the three separate focus group sessions (Additional file 2). Five researchers from the IFIP project group (KMH, MR, RP, LH, and KSH) conducted the data collection at the CMHCs, working in pairs of two interviewers at each focus group. The focus groups lasted for 60–90 minutes and were audio recorded. All participants were informed about the study and gave written consent to participate. To become immersed with the data, and to adjust the interview guides according to new emerging themes, the interviewers wrote a brief report with important highlights immediately after each focus group. Scientific assistants and other project members transcribed the interviews verbatim. All data-material were stored in the University of Oslo’s secure database (In Norwegian: “Tjenester for sensitive data”–TSD).

### Analysis

The first author (KMH) performed the main analytical work. This sub-study is a follow-up on an overarching barrier-facilitator study [42], thus, the overall analysis of barriers and facilitators was extended by a separate in depth-analysis of ethical challenges, barriers, and possible solutions regarding confidentiality. The manifest content analysis [47] progressed through three main phases: 1) The preparation phase, which involved a thorough reading of the transcripts to become immersed in the data and obtain a sense of the whole, 2) The organising phase that involved the initial coding and categorising of the transcripts, and 3) The reporting phase in which a repeated abstraction process led to the identification of five themes describing ethical challenges and two themes describing measures to handle such challenges, in addition to making defensible links between the data and the results through the presentation of relevant quotes.

Data storage and the analytical work was performed with the NVivo computer software package 12. In the following we present the findings partly as condensed text [48] and partly as illustrative quotes. Our focus was to render the meaning content of the participants’ accounts, thus the quotes are condensed.

### Credibility and transferability

Various triangulation strategies increased the trustworthiness of the study’s findings. To develop a comprehensive understanding of the phenomenon under investigation [49], health professionals with various professional background, experiences and roles in the implementation work were included in the study (data source triangulation). The study design further enabled an exploration of participants’ perspectives and experiences with confidentiality issues over time, which provided us with knowledge on important change processes. Further credibility was established by seeking agreement among co-researchers [50] in which KMH, MR, and RP discussed the data labelling and the grouping of themes and subthemes in repeated sessions. Furthermore, members of the research group (KMH, MR, KSH, LH, BW, EL and RP) engaged in discussions about preliminary findings and contributed with drafts revisions during the writing process. Finally, the credibility of the findings is enhanced by presentation of the findings together with rich and representative quotations that show the similarities within and differences between categories [50] and provide details and contextual information.

## Results

The results are presented in two parts, Part 1: “Ethical challenges related to the duty of confidentiality” and Part 2: “Measures to facilitate better handling of the duty of confidentiality”. Data on facilitating measures derived from participants who had experiences with family involvement as part of the IFIP trial, as well as from participants with extensive experience with family involvement prior to the trial.

Data from the initial phase of implementation (Fig. 1) demonstrate a wide range of understandings, practices, and challenges related to confidentiality. These variations often represented disagreement on how to handle various aspects of the duty of confidentiality. Rather than ethical challenges, some of these may be best described as unreflected attitudes or practices where the duty of confidentiality became an absolute barrier to family involvement. These variations and uncertainties were often due to a lack of knowledge, e.g. about the legal regulations, and lack of knowledge is not an ethical challenge. However, not all types of uncertainties were due to lack of knowledge, and there were disagreements on what to do when facing such uncertainties.

### Part 1: Ethical challenges related to the duty of confidentiality

#### Uncertainty in how to apply the legislation

Fear of breaching the legal duty of confidentiality was described by many professionals as a major barrier to family involvement:

*The duty of confidentiality is perhaps the greatest problem when working with relatives. It is what hinders us the most (…) People are very afraid of doing anything wrong (FG6).*

Accounts also demonstrated that the duty of confidentiality appeared difficult to understand and to transfer to clinical practice. Some participants interpreted the legislation very strictly, thus refraining from family involvement, while others saw possibilities to make their own clinical judgments:

*On a national level, I imagine that the law itself can be a bit clearer (…) There are a lot of “gray areas” and a lot of ... yes, uncertainty. If you ask ten different health professionals, you get ten different answers to what is okay to say and what is not right (FG6).*

Some participants also admitted that they had been hiding behind the duty of confidentiality in order to “solve” demanding situations.

#### Patient autonomy versus a less strict interpretation of the duty of confidentiality

Ethical challenges occurred when participants tried to initiate family involvement but were faced with patients not consenting to involve/disclose information, when patients occasionally gave and withdrew consent, or when they suddenly changed who should be listed as their next of kin. Such situations were experienced as particularly challenging and caused doubt and uncertainty in terms of “What is the right thing to do?” Several were unsure whether and how they could engage and communicate with the relatives if the patient refused any contact, or if consent was not clarified, and they dealt with refusals quite differently:

*(You) can’t just call people, I think, if you haven’t received consent (FG7).*

Another clinician chose to oppose the patient and legislation by contacting the relatives despite the refusal:

*(…) the patient was adamant that the relatives should not be involved. And the relatives were extremely worried. With good reason (…) This is a typical situation, and sometimes we do say “In this case I choose to inform your relatives even if you deny it”. But, the threshold is high (FG1).*

Even if the participants recognised the benefits of family involvement for the patients and the relatives, most respected the patient’s refusal and decided not to challenge the lack of consent any further. According to some participants this could be due to strong patients’ rights regarding confidentiality that they felt had to be fulfilled or fearing negative reactions leading to patient autonomy triumphing over other concerns:

*When resistance arises, one withdraws very quickly. Avoids it. You somehow do not feel that you have anything to offer. You know, there are many paranoid patients and relatives...(FG6).*

Participants also shared their opinions on why patients refuse, for example, that they rejected family involvement to avoid burdening their family:

*I am surprised (…) she (the patient) is quite ill, has been ill for many years, and then I suddenly was thinking… did we lose grip of him? I mean... she does have a boyfriend… “Does he know how you feel?” “No, she didn’t want to burden him” (FG8).*

Other patients were sceptical and ambivalent to involving their family due to lack of control, and the fear that clinicians would disclose sensitive information:

*Several patients are very skeptical that I should talk to their relatives because… they have an understanding of, or have thoughts that I’m going to disclose… how much hashish they have smoked (FG6).*

#### Patient alliance and beneficence versus a strict interpretation of the duty of confidentiality

Some participants noted that they accepted refusals because they worried that contacting the relatives or divulging any kind of information would damage patient trust, thus potentially threatening the therapeutical alliance that they considered crucial to enable appropriate treatment:

*… I am concerned that the patient will reject home visits (having contact). That the therapeutical alliance can slip if one pushes too hard (FG14).*

Other participants expressed concerns that a disproportionate/excessive emphasis on the patient alliance could lead to professionals losing the alliance with relatives:

*The first thing the relatives are saying is that they constantly are met by a “wall of confidentiality”, thus they receive no information. And this creates a lot of despair, right, a feeling of not being seen. You somehow feel that the duty of confidentiality is just for the professionals so they can avoid doing a job (FG3).*

Some weighed the need to maintain the patient alliance against the possible benefits for patients of involving their family. By accepting the refusal, they were aware that they lost access to a potentially important treatment resource and to improve or sustain the patient’s social network.

#### How to deal with uncertainty regarding what relatives know about the patient’s illness

Managing information disclosure was experienced challenging when family involvement and family relations had not yet been discussed and clarified with the patient:

*It can be a bit difficult sometimes when the relatives are calling; What kind of information have they received earlier? What can we say? I’m not sure whether we always document these phone-calls (FG2).*

Another typical situation where this ethical challenge emerged was when the participants wanted to get in contact with a relative for the first time, for example, to improve the medical investigation:

*P1: But obviously, if you believe that the relatives know that the patient is here (at the hospital)...?*

*P2: But how can I know if they have not made any contact? (FG7).*

At the initial treatment stage when contact was not yet established between the family and the services, uncertainty as to how one should operationalise the duty of confidentiality and the informed consent disrupted the onset of family involvement.

#### Relatives’ interests versus the duty of confidentiality

Even if patient autonomy mostly triumphed other concerns, participants were repeatedly faced with stakeholders’ (seemingly) diverging needs, for example, balancing patients’ need for privacy against relatives’ interests and their legal right to being informed and involved. Typical challenging situations arose when participants considered it important for relatives to receive support and information about the patient while the patient refused:

*To receive consent that the relatives can gain some insight is one of the biggest challenges. The patient spends a lot of time “keeping people away” (…) being healthy in the eyes of the relatives. And the relatives are screaming for information (…) Through many years as relatives, there are many who certainly have not received information and who feel quite helpless (FG1).*

### Part 2: Measures to facilitate better handling of the duty of confidentiality

#### Training and practice in family involvement

The most important measure to facilitate better handling of the duty of confidentiality seemed to be training in family involvement followed by practice. The new theoretical and experience-based competence, specific skills, and positive experiences with family involvement made the participants better equipped to deal with the ethical challenges.

##### Increased understanding of the significance of family involvement

The participants stated that with increased competence and experience they became more aware of the significance of family involvement to improve treatment, help patients sustain core relationships, and support their families:

*When relatives lack information and feel excluded from collaboration, I experience that many are very anxious and in some cases are calling us extensively. But if you take your time and talk and listen to them, and perhaps arrange for a joint conversation with patient and relatives, things calm down for them. And of course for the patient (FG16).*

Increased understanding of the significance, consequences, and alternative solutions further led to a change in how participants managed the engagement phase.

##### Improved strategies for approaching and informing the patients

Learning how to exercise the duty of confidentiality and fulfil relatives’ legal rights, while also feeling confident that their efforts to integrate family members in the treatment most likely would benefit the patient, made participants more self-confident when asking patients for permission to contact or disclose information to their relatives. Several participants informed their patients about their relatives’ rights to information and support, and asked questions like: “How can we best tailor family involvement to your needs and concerns?” When participants approached the patients with thorough, attentive conversations about family involvement, trust and understanding increased among patients that openness towards their family could be helpful to all parties. Participants’ accounts demonstrate how they successfully obtained consent by informing, assuring, and motivating the patient:

*Many patients experience pressure from their relatives, a lot of expectations, demands and criticism. However, providing relatives with thorough information can actually alleviate that pressure. It is very important to take this approach because it can solve a number of such situations where the patient does not want (family involvement). Further I believe that for some of our patients it is important that they know that their relatives can talk to us and get some relief. It helps them, and in the end it helps the patient (FG11).*

To overcome distrust and scepticism, participants assured the patient that no sensitive information would be disclosed without their consent:

*P1: Often it is useful to make an agreement with the patient about what information will be disclosed so that the patient knows – and has accepted – what is being said to the relatives.*

*P2: We make clear that “We do not inform relatives about this and that, and they rarely want to know this and that” - it’s more like “What will happen in the future, what kind of treatment the patient receives, what is the prognosis?” (FG4).*

When consent and mutual agreements were obtained, participants could provide the relatives with information about the state and treatment of their loved one. Several participants provided general information about the diagnosis, if known to the relatives, and they asked about what relatives already knew and thematised this further. Providing support and guidance to improve relatives’ coping with their own situation and to optimise patient support was not only emphasised as crucial to the relatives, but also constituted meaningful clinical encounters for the participants. Their motivation to continue their efforts to balance the duty of confidentiality against other concerns increased when experiencing the significance of providing even limited information to the relatives.

##### Improved strategies for dealing with patient refusal

Several participants experienced situations where patients refused to share information despite initiatives to increase trust. Those who knew how to differentiate the various types of information managed to meet the relatives’ needs without breaching confidentiality:

*It is important that both relatives and the patient are aware of this, that even if the patients don’t want us to talk to the relatives, they actually have a right to receive information both about the treatment and psychosis in general. Understanding this was very “clarifying” to me, because this is what we have been struggling with all these years, and this has made us refrain from talking to relatives… (FG10).*

Suddenly, when encountering the relatives, the focus shifted from disclosing patient information to active listening and providing less sensitive, but at the same time tailored information:

*… But in fact we should turn it around; we should “hear them out”, we should investigate and the things they share, we can say something about this on a general basis (FG6).*

Some started to consider obtaining consent to be a stepwise process, which required patience, sincere recognition of patients’ concerns, and explorations of possible reasons underlying the refusals:

*I can ask the patient: “Why don’t you want to talk about it?” (…) Is it shame, are they afraid that the parents will be worried, upset, (that they) will inflict on them something unpleasant - are there such thoughts? (FG12).*

An interesting finding was how some participants met the patients with completely different determination and stamina when suggesting family involvement than they had before the implementation:

*Now, when the patient refuses family involvement, I have been even tougher to listen to relatives about known knowledge. And talk to them (FG5).*

##### Facilitating semi-open triadic dialogues

When one managed to arrange conversations with the patient, relatives, and the therapist together, dealing with confidentiality was experienced as less challenging. The need for keeping things confidential decreased as a result of trust, openness, and shared understanding between stakeholders:

*Confidentiality is generally a challenging topic (…), but when it comes to relatives and patients agreeing on a (family psychoeducation) group (…) there is usually no big problem with confidentiality because then they have received good information, then we have built up an alliance… and they have received knowledge. There is agreement on cooperation, but that does not mean that we can just pour out… everything somehow. The patient must always feel confident that their interests are the number one priority (FG19).*

#### Standardisation of family involvement practices

The focus group interviews demonstrated an explicit need for standardisation. In particular, the initial engagement phase seemed to constitute a recurring weakness in the units’ family involvement practices. Procedures that support professionals in navigating confidentiality during this initial phase were welcomed by participants:

*We have such a reception note that everyone must make at the first call / reception. The relatives are a separate point, same as for suicide risk, right (…) there you get something like “Who is your closest relative?”, but in extension of that - how to talk about that collaboration? (FG7).*

Others considered standardisation to be a means to increase the legitimacy of family involvement:

*It is easier for the patient to say yes to something that is known to be part of the standard package here (FG14).*

Furthermore, participants voiced a need for systematic training of professionals:

*We simply need a lot of professional development in how to work with patients to motivate them to give consent (FG3).*

A key IFIP intervention measure was to offer early and standardised conversations about family involvement to all patients and relatives as a default approach [40]. The significance of such routines was appreciated by several participants:

*(…) if one succeeds, then things are “put down” quite early (…) If you can get it done relatively early, then things can be shared, you can talk to each other without anything building up (FG5).*

## Discussion

Within the frames of an implementation study, we explored what ethical challenges and facilitating measures mental health professionals in CMHCs experienced related to the duty of confidentiality regarding family involvement for persons with severe mental illness. Key ethical challenges identified were how to balance patient autonomy versus a less strict interpretation of the duty of confidentiality, how to balance patient alliance and beneficence versus a less strict interpretation of the duty of confidentiality, dealing with uncertainty when one does not know what the relatives know about the patient’s illness, and how to balance the best interest of the relatives versus keeping patient information confidential. In addition, we found that participants’ lack of knowledge on how to apply the legislation constituted an absolute barrier to family involvement in some cases. How participants dealt with a lack of consent or explicit patient refusals in the initial phase of family involvement appeared critical to the integration of the family in treatment and care.

Nevertheless, our findings clearly showed that there are ways to improve the handling of these ethical challenges. The key measures were training in family involvement followed by practice and standardisation. When participants gained competence in confidentiality statutes, in how to thematise confidentiality with patients and relatives, and how to perform recommended family involvement, most challenges were experienced as solvable.

### Dealing better with ethical challenges by reframing the duty of confidentiality

We hypothesise that the improvements that took place in this study occurred through a reframing of the duty of confidentiality. This reframing can be understood as a change in interpretation and practice with regard to the legislation, a change in ethical reasoning, and a change in clinical practice.

#### A change in interpretation and practice with regard to the legislation

First, reframing the duty of confidentiality represents a “move away from simplistic rules about confidentiality” [29] towards practicing the legislation more flexibly (and legally correctly). In line with previous research [22], we found that confidentiality issues raised from a strong focus on and incorrect interpretation of legal matters. The overarching question of family involvement was often erroneously and too closely linked to the distinct question of disclosing information. Challenges arose because the professionals lacked the necessary understanding of a) how to differentiate between general information, which can be shared without consent, and personal information requiring consent because it involves new and specific information about the patient [15, 51], and b) how disclosing personal information about patients to third parties is seldom necessary to perform the recommended family involvement, nor is this what the relatives commonly demand [51]. While implementation progressed, participants increasingly managed to take various considerations and needs into account. Furthermore, information disclosure was to a greater extent experienced as a means to establish contact with family and to enable good treatment, rather than being a troublesome “obstacle”.

#### A change in ethical reasoning

Second, reframing the duty of confidentiality denotes a changed weighting of autonomy against the other three basic ethical principles of beneficence (what would be beneficial to the patient (and/or their relatives?), non-maleficence (does accepting the refusal outweigh the potential harm to the patient and/or their relatives?), and justice (can accepting this refusal be justified with regard to the relatives?). Before the implementation, the participants tended to accept patient refusals and prioritised patient interests/autonomy and the therapeutic alliance [18, 52]. During implementation, however, participants experienced that most of the diverging needs appeared reconcilable or that other concerns appeared to be equally important. With that, changed their ethical reasoning.

An interesting finding from this study is how some participants in their quest to protect patient autonomy unintentionally – and paradoxically – undermined autonomy by not providing a real basis for decision-making. When professionals do not ensure that refusal to involve the family is given on an informed basis, this may be described as “the duty of confidentiality paradox”. To understand what the consent entails, patients are dependent on professionals to provide sufficient and tailored information about how the family can contribute to treatment and receive support, why this is important, possible side-effects and how to deal with them, that the information that needs to be shared is by and large general and not sensitive, and that collaboration and information disclosure can be tailored to both the patients’ preferences and the relatives’ needs. Only then patients are able to make an autonomous choice whether and how they want to involve their family.

Prior to implementation, several participants accepted refusals without having the necessary competence to a) provide the above-mentioned information, b) explore what the refusal entailed specifically and whether the patient was aware of its consequences, c) assess the advantages and disadvantages of involvement, and d) identify alternative solutions (e.g. to further thematise family involvement when the disease state is improving). Issues arose because the patients lacked the necessary understanding, which is required for autonomous actions [36]. Therefore, professionals should not accept a refusal too quickly because they need to understand the worries and values that underlie this refusal. During the IFIP trial, several participants shared experiences where patients refused involvement in order to spare their parents from burdens and worries, while the parents suffered greatly from being excluded from crucial life events of their child battling severe mental illness.

The “inflation” of the duty of confidentiality and “the duty of confidentiality paradox” constitute severe errors with severe consequences. Our data contain repeated descriptions of patient refusals leading to poor, interrupted, or absent family involvement and support. First, this leads to patients with varyingly impaired capacity being left alone and unenlightened when making critical decisions regarding treatment methods and support. Thus, the issue of (ambivalent and unsure) patients refusing to involve their closest relations is sustained. Second, a one-sided focus on patient alliance and autonomy comes at the expense of professionals losing the alliance with the relatives [15], which might further negatively impact the patient-relative alliance. Yet another problem is that patient refusals hinder family involvement before one has had the chance to experience the benefits of such collaborations. Several participants shared positive experiences from engaging with the families, for instance that they gained useful collateral information about the patient or realised how the family dynamics improved.

#### A change in clinical practice

**Fig. 2:**
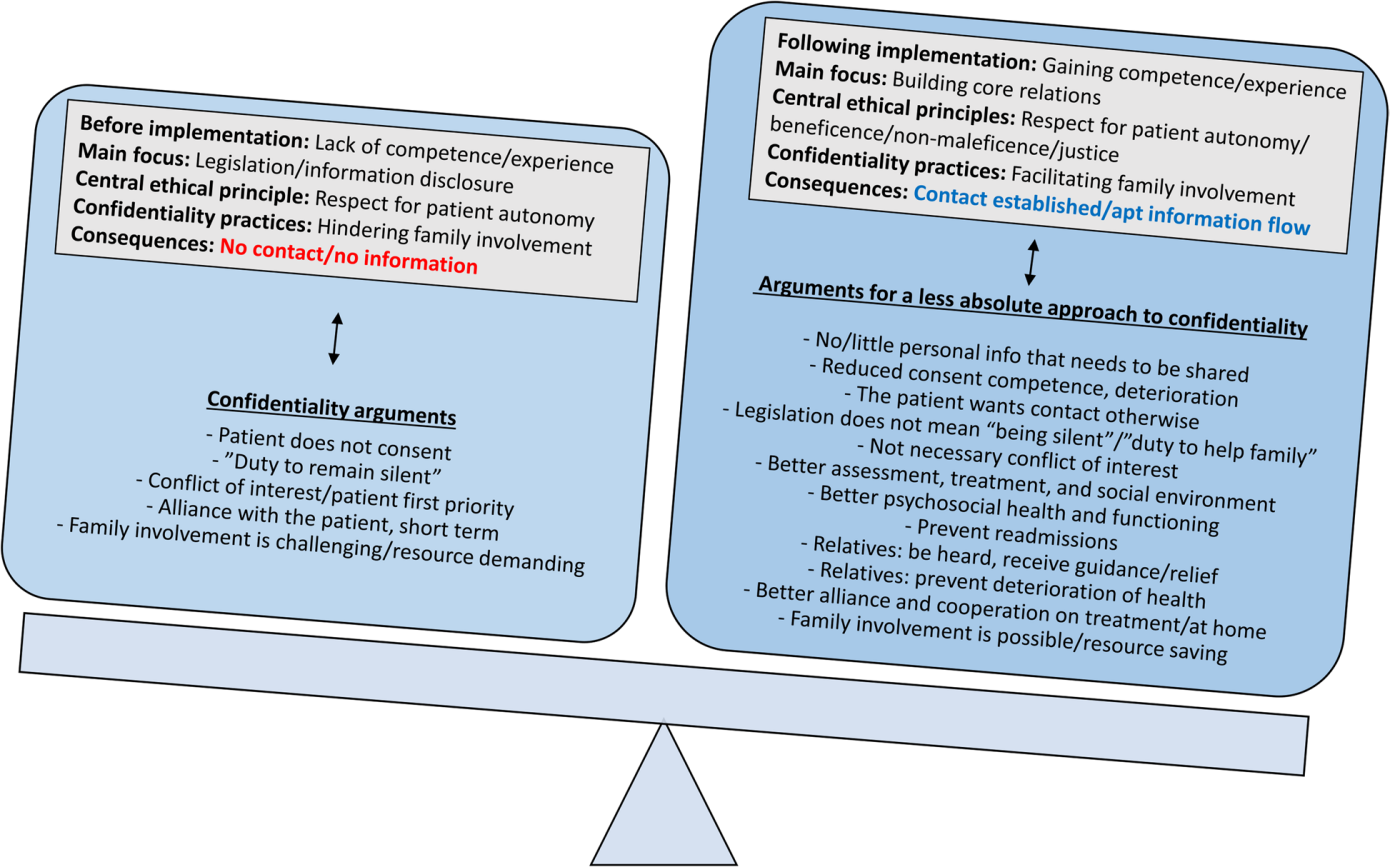
Changes that improved the handling of the duty of confidentiality

Finally, reframing the duty of confidentiality requires new and more appropriate clinical practices, such as routinely initiating dialogues with patients at an early stage, tailoring disclosure to the individual’s family [32, 33], asking patients how they want family members to be involved [53], and implementing the use of “disclosure to consent” forms [16]. Further measures were to handle refusals more appropriately, for example, by further investigating the reasons for refusing, considering consent to be a stepwise process [27], and distinguishing between general and specific information [51]. Finally, consciousness was raised on relatives’ own issues and on what one can offer the relatives when patients refuse [18]. Figure 2 summarises the changes that occurred during the IFIP implementation.

In line with previous studies, we found that the implementation of sound confidentiality practices presupposes the implementation of clear procedures for obtaining consent and for releasing information to families [29], in addition to familiarising clinical staff with family interventions, relevant legislation and mental health policies [15, 18, 20, 26]. A more overarching cultural shift in mental health care, including a change in attitudes towards working with families [15, 20, 32], is needed to facilitate the above-mentioned measures.

### Unresolved legislative barrier

Although most ethical challenges can be diminished through competence measures, a residual legislative problem remains. As in many other countries, including England and Netherlands, Norwegian health legislation does not allow disclosure of even a minimum of information without consent (with a few exceptions) to relatives of competent adult patients. It is not an exception that the relative as “significant others” engage in a close relationship with the patient and provide essential daily care. One example of unlawful disclosure of minimum information is when a worried mother approaches the services inquiring about her sons’ condition and receives a confirmation that he is ok and currently taken care of. The issue arises because the health legislation does not differentiate the degree of sensitivity of health information as long as it is can be linked to the patient. Thus, there is little room for discretion or for considerations of proportionality. This is contrary to, for example, the General Data Protection Regulation (GDPR) [54], where proportionality between privacy concerns and other interests, such as patient safety and the relatives’ interests, is encouraged. Even if the information disclosed in the above-mentioned situations can be considered the least sensitive, the law applies the same as if the nurse were to share with the mother the entire patient record. This legislation with regard to relatives appears inappropriately limiting. We encourage minor legal changes to be made in order to allow mental health professionals, in certain situations where competent patients do not consent, to share a minimum of patient information with the relatives. The benefits of meeting the mothers’ request, might be argued to outweigh the minimal damage inflicted on the patient.

### Strengths and limitations

The main strengths of this study are the study design and the ongoing evaluation that enabled rich data on professionals’ experiences with confidentiality issues over time. Most of the authors of this paper have been deeply involved in all aspects of the IFIP implementation/research from which this article emanates and possess a broad expertise relevant to probing into the complexity of confidentiality. This further strengthens the credibility of the findings. Limitations include the lack of patients and relatives’ perspectives, thus it has not been possible to compare perspectives and experiences. Furthermore, this study was conducted within the frames of a distinct implementation effort, thus the training and implementation support provided may have influenced the results presented in this paper. In terms of generalisability, the scope of this study is limited to competent patients who suffer from psychotic disorders and who receive treatment in Norwegian CMHCs. Nevertheless, we might assume that the ethical challenges and facilitating measures identified in this study are relevant to other clinical settings.

## Conclusions

Confidentiality issues are among the most prominent barriers to family involvement during severe mental illness, with mental health professionals facing complex ethical, legal, and practical challenges. Within the current implementation study, clinicians struggled with how to apply the legislation, how to balance patient autonomy, alliance, and beneficence with a less strict interpretation of the duty of confidentiality, and how to balance the best interest of the relatives with keeping patient information confidential. Training in family involvement, followed by practice, led to a vital change in how clinicians approached the patients when initiating family involvement, how they dealt with patient refusals, and how they valued and interacted with the families. To achieve such improved confidentiality practices standardisation of family involvement, implementation of confidentiality guidelines, and incorporating basic training in family involvement in the health educational institutions/services is required. An attitudinal, organisational, educational, and legal shift in terms of how clinicians relate to confidentiality and how they value informal care is essential to facilitate the integration of families as both collaborative partners and carers with their own sufferings and needs.

**Table:** 

**Highlights for clinical practice** • The duty of confidentiality is challenging and complex in family involvement for persons with severe mental illness, and is often interpreted too strictly as requiring “total silence”. • Relatives have the right to general, as well as known information, training, and support, even without the patient’s consent. • One can listen to and provide support to relatives without the patient’s consent. • Family involvement should be discussed with all patients as a default approach. • Training and guidance in family involvement for professionals, followed by practice, improves confidentiality practices within mental health care.	

## Supplementary Information

**Additional file 1.** Standards for Reporting Qualitative Research (SRQR)*.

**Additional file 2.** Interview guides.

## Data Availability

The datasets used and/or analysed during the current study are available from the corresponding author on reasonable request.

## Abbreviations

IFIP: Implementation of guidelines on Family Involvement for persons with Psychotic disorders in community mental health centres
CMHC: Community Mental Health Centre
FPE: Family Psychoeducation
TSD: Tjenester for Sensitive Data
GDPR: General Data Protection Regulation
